# Serum bile acid profiles in pediatric gastrointestinal, hepatic and biliary diseases

**DOI:** 10.1186/s40348-025-00211-2

**Published:** 2025-11-28

**Authors:** Katja Linz, Felix Wachter, Merle Claßen, Jakob Zierk, Theresa Voggenreiter, Alexander Schnell, Henriette Grieshaber Bouyer Mandelbaum, Joachim Woelfle, Ferdinand Knieling, André Hoerning, Manfred Rauh, Adrian P. Regensburger

**Affiliations:** Department of Pediatrics and Adolescent Medicine, Friedrich-Alexander- University (FAU) Erlangen-Nürnberg, Loschgestr. 15, Erlangen, 91054 Germany

**Keywords:** Bile acids, Gastroenterology, Hepatology, Biliary diseases, Pediatrics

## Abstract

**Background and aims:**

Altered bile acids (BA) are key drivers of hepatic disorders and beyond. The breakdown of BA profiles could serve as advanced biomarkers, but data in pediatric patients is scarce. In this work we retrospectively analyzed routine BA profiles of various pediatric gastrointestinal, hepatic and biliary diseases.

**Methods:**

Routine tandem mass spectrometry derived serum BA profiles from a ten-year period (2014–2024) of a pediatric tertiary care center were analyzed. First, guidance values for 15 bile acid components for six age groups were established. Next, BA profiles were examined across gastrointestinal, hepatic and biliary diseases.

**Results:**

A total of 1906 bile acid profiles from *n* = 524 patients were analyzed, with 1341 (*n* = 167) profiles from patients with gastrointestinal, 222 (*n* = 143) with hepatic, and 269 (*n* = 159) with biliary diseases. Total primary BA were found to be higher in younger compared to older children, with an increase in secondary BA with age. Significant BA alterations were observed in patients with certain hepatic and biliary diseases including an increased primary to secondary BA ratio, a reduced proportion of unconjugated BA and increased glycine- to taurine-conjugation ratio.

**Conclusion:**

We provide guidance BA profile values for different pediatric age groups and an overview of altered BA profiles in gastrointestinal, hepatic and biliary diseases. This work emphasizes the potential of BA profiles as a diagnostic tool and could serve as a guide for clinical interpretation and as framework for future studies in the field.

**Supplementary Information:**

The online version contains supplementary material available at 10.1186/s40348-025-00211-2.

## Background

Once primarily recognized for their function as detergents for fatty acids and vitamins, bile acids are now understood to have complex endocrine and metabolic function [1]. Several bile acid receptors regulate the glucose and lipid metabolism, the energy homeostasis, cell proliferation and inflammation [2]. Furthermore, BA composition is highly influenced by the gut microbiota [3] and plays a decisive role in the carcinogenesis of colorectal cancer and hepatocellular carcinoma [4]. This signaling and central regulatory position makes BA essential in maintaining physiological gastrointestinal and hepatic metabolism and immune homeostasis [5]. BA are synthesized in the liver from cholesterol producing two primary BA (cholic acid and chenodeoxycholic acid), which are then partially conjugated with glycine or taurine before being secreted into the bile ducts and duodenum. In the intestine, bacterial enzymes transform primary BA through deconjugation and dehydroxylation, resulting in the secondary BA. The majority of BA are then reabsorbed via the enterohepatic circulation [6, 7]. Given their pivotal role in endocrinology and metabolism, BA metabolites hold significant diagnostic potential for providing deeper insights into microbiome alterations and for diagnosing various liver, biliary and intestinal diseases. Advances in laboratory medicine, particularly mass spectrometry, now enable the detailed analysis of multiple BA in the blood, which has enhanced our ability to identify BA patterns as biomarkers of disease [8, 9]. For instance, specific BA profiles have been used to monitor disease activity in pediatric patients with inflammatory bowel disease [10]. The link becomes even more evident in pediatric cholestatic liver disease, where bile homeostasis is corrupted and the leading reason for liver transplant [11]. Information about specific BA concentrations revealed prognostic relevance in e.g., patients with Alagille syndrome [12]. Furthermore, novel therapies to modify the BA pool by inhibition of the ileal BA transporter newly exist for infants with Alagille syndrome and progressive familiar intrahepatic cholestasis to reduce the torturing pruritus [13–15]. Despite the critical role of BA in pediatric hepatology, reference values and comprehensive BA profiles for children are still limited. In this retrospective study we analyzed BA profiles routinely collected over a ten-year period in our excellence center of mass spectrometry with regard to pediatric gastrointestinal, hepatic and biliary diseases.

## Methods

### Aim of the study and study design

Bile acid profiles are emerging as promising biomarkers that can support clinical decision-making. However, there is a lack of published data demonstrating how alterations in bile acid metabolism are reflected in bile acid profiles across various pediatric diseases that affect this pathway. The aim of this study is to illustrate how bile acid profiles can be altered in a variety of diseases affecting bile acid metabolism, and thereby providing clinicians comprehensive data to aid interpretation of their measurements.

Therefore, a retrospective study was conducted to analyze BA profiles from in- and outpatients of a tertiary care center for children and adolescents. All BA profiles were analyzed by the same local center for mass spectrometry in routine care. Results from patient samples from October 2014 to April 2024 were used for this study. The study was approved by the local ethics committee (Nr. 24–92-Br).

### Data retrieval and processing

The inhouse data warehouse was searched for pediatric patients with BA profiles in the medical records. Serum BA profiles consist of liquid chromatography–tandem mass spectrometry (LC–MS/MS) measurements of primary (CA = cholic acid, CDCA = chenodeoxycholic acid and their different G = Glycine-, T = Taurine- conjugated forms), secondary (DCA = deoxycholic acid, LCA = lithocholic acid and their G- and T- conjugated forms), and tertiary BA (UDCA = ursodeoxycholic acid and its G- and T- conjugated form). Furthermore, the measured BA were used to calculate additional absolute values and ratios. This included total BA, total primary, secondary and tertiary BA, total unconjugated, conjugated, glycine-conjugated and taurine-conjugated BA and their respective proportions plus a primary to secondary BA ratio and a ratio of glycine- to taurine-conjugated BA.

Associated laboratory parameters, patient demographics and diagnoses were retrieved accordingly. For each hospital visit, all ICD diagnoses were recorded, and the diagnosis most likely linked to the BA profile testing was designated as the primary diagnosis for group comparisons. The three main groups were gastrointestinal, hepatic and biliary diseases with respective subgroups (e.g., Crohn´s disease, liver failure, primary sclerosing cholangitis, etc.).

### Derivation of guidance values

To assess the measurement values of the patient groups, guidance values were established to provide orientation. These were derived in the following manner: Every sample underwent a filtering process consisting of two steps: First a threshold-based filter was applied to all samples to eliminate BA profiles with increased (considered pathological) total BA. The cut-off value for filtering originates from adding three standard deviations to the mean value of a supervised healthy population with age-corrected reference intervals provided by Jahnel et al. [16]. We further added a filter on total tertiary BA in order to eliminate samples who were treated with ursodeoxycholic acid. This resulted in a sample size for the guidance values of *n* = 1471 from 354 patients that were further divided into age groups from 0 to 5 months (*n* = 11), 6–23 months (*n* = 30), 2–5 years (*n* = 34), 6–11 years (*n* = 284), 12–19 years (*n* = 1082) and above 19 years (*n* = 30). We then used 97.5% and 2.5% percentiles as guidance values.

To validate our approach, we compared the guidance values with those generated by the established refineR algorithm [17], which derives reference intervals from real-world data (and therefore does not require a healthy reference population). To this end, we tested whether the generated guidance values fall within the 95% confidence intervals of the upper reference limits established using refineR.

### Comparison of bile acid profiles in different diseases

To generate bile acid profiles for different diseases, we used the entire dataset without applying any filters. An average value was first calculated for patients that provided multiple samples. A median was then determined across all patients within each group. This median was subsequently used for comparisons with our derived guidance values as described above.

### Statistics

Descriptive data are given as median and IQR or number and percentage. Orientation values are given as median with the 2.5% and 97.5% percentile. Correlations are given as Spearman correlation coefficient. Kruskal-Wallis test with Dunn comparison was used to test for significance. All calculations were performed with GraphPad Prism (Version 10, GraphPad Software, Boston, Massachusetts USA).

## Results

**Fig. 1:**
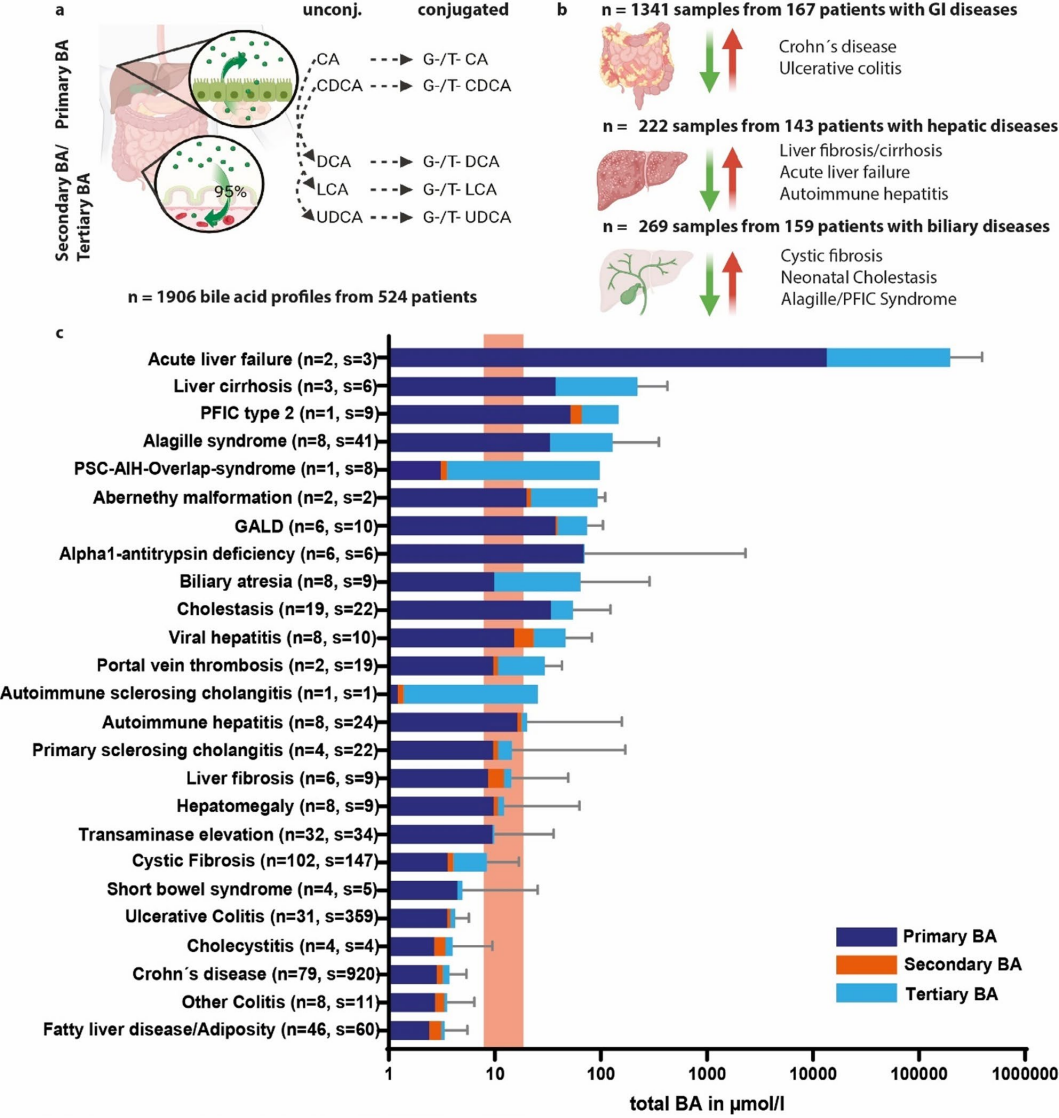
Study procedure, population and total bile acid abundance. CA = cholic acid, CDCA = chenodeoxycholic acid, DCA = deoxycholic acid, LCA = lithocholic acid, UDCA = ursodeoxycholic acid, G = Glycine-, T = Taurine-, n = number of patients, s = number of samples. **a** Liquid chromatography–tandem mass spectrometry (LC–MS/MS) of 15 BA composites (unconjugated and conjugated primary, secondary and tertiary BA) was performed routinely in the tertiary care center. **b** Patients were assigned to gastrointestinal, hepatic or biliary as main disease group. These main groups were than further divided for the respective main diagnoses. **c** Different diagnoses from gastrointestinal, hepatic and biliary diseases are ordered by the height of total BA elevation. Displayed is the median and interquartile range of the total bile acids of every group. The proportion of primary (dark blue), secondary (orange) and tertiary (light blue) BA is labeled, accordingly. The redish area shows the range of cut-off values for the different age groups. Created with BioRender.com, Graphpad Prism and Adobe Illustrator software.

In this study 1906 BA profiles from 524 patients (with 1–53 samples) from routine patient care were analyzed (Fig. 1a). 176 patients had 2 or more samples (median 4, range 2–53). The patients had a median age of 13 years (IQR = 5.4–16.3). 234 (44.7%) patients were female.

Three main groups were analyzed with 167 (31.8%) gastrointestinal patients and 1341 samples, 143 (27.3%) hepatic patients and 220 samples and 159 (30.3%) biliary patients and 269 samples, respectively (Fig. 1b). 55 (10.5%) patients and 76 samples were classified as “other” with varying diseases from e.g., hematology. The numbers of each diagnosis within each main group are displayed in Supplementary Table 1 (e.g., Crohn’s disease with 920 samples in 79 patients). Demographic characteristics and routine laboratory parameters (e.g., bilirubin, GOT) are given in Table 1.

**Table 1:** Demographics and standard laboratory parameters

	Gastrointestinal dis. *n* = 1341 samples in 167 patients	Hepatic diseases *n* = 220 samples in 143 patients	Biliary diseases *n* = 269 samples in 159 patients	Others *n* = 76 samples in 55 patients
Age (years)	14 (12–16)	11 (5–15)	14 (4–23)	4.5 (0–13.25.25)
Gender	f = 591 (= 44.1%), m = 750 (= 55.9%)	f = 88 (= 40%), m = 132 (= 60%)	f = 115 (= 42.8%), m = 154 (57.2%)	f = 33 (= 43.4%) m = 43 (= 56.6%)
T BA (µmol/l)	3.055 (1.736–5.261)	10.571 (4.029–43.013)	15.230 (5.532–54.620)	12.704 (3.481–55.908)
GPT/ALT (U/l)	15 (11–20)	66 (34–115)	31 (20–70)	28 (15–78)
GOT/AST (U/l)	21 (18–25)	53 (34–79)	30 (22–56)	47 (27–84)
gGT (U/l)	15 (12–19)	35 (19–81)	28 (15–154)	34 (14–220)
dBili (mg/dl)	0.10 (0.10–0.20)	0.20 (0.20–0.40)	0.30 (0.20–0.63)	0.70 (0.20–6.50)
AP (U/l)	132 (86–203)	236.5 (125–345.75.75)	216 (121–379)	283 (181–545)

*T BA* total bile acids (serum), *GPT/ALT* glutamate-pyruvate transaminase/alanin aminotransferase, *GOT/AST* glutamic oxaloacetic transaminase/aspartate aminotransferase, *gGT* gamma-glutamyltransferase, *dBili* direct bilirubin, *AP* alkaline phosphatase, *f* female, *m* male

Descriptives are given as median with interquartile range (IQR) or number with percentage (%).

### Decoding ursodeoxycholic acid therapy by bile acid profiles

To filter patients treated with ursodeoxycholic acid (UDCA) by their BA profile, 200 patients were randomly selected from our cohort and divided into two groups. Group 1 consisted of 94 patients without UDCA therapy and Group 2 of 106 patients treated with UDCA. As a surrogate marker for UDCA treatment, the total count of tertiary BA demonstrated the best diagnostic performance, with an area under the curve (AUC) of 0.96 (*p* < 0.0001) compared to 0.87 for the absolute count of UDCA and 0.87 for the proportion of tertiary BA. By applying a cut-off value of 2.322 µmol/l, it is possible to distinguish between the two groups with a sensitivity of 95.28% and a specificity of 95.74%. According to this cut-off, 272 of all our samples were classified as UDCA recipients, whereas 1636 samples were not classified as such.

### Guidance values of bile acid profiles

The cohort was divided into six age groups and orientation values were calculated as median, 2.5 and 97.5 percentile for each of the 15 BA and corresponding compositions and ratios (Table 2). The range in sample size with lower number of samples for chenodeoxycholic acid and deoxycholic acid was attributed to the interfering of the BA with CFTR-potentiators e.g., Ivacaftor, given to patients with Cystic Fibrosis [17].

**Table 2:** Guidance values for bile acid derivates and calculated proportions and ratios

	0–5 months			6–23 months			2–5 years			6–11 years			12–19 years			> 19 years		
	(*n* = 11)			(*n* = 29–30)			(*n* = 31–34)			(*n* = 271–284)			(*n* = 1025–1082)			(*n* = 5–30)		
	median	2.5-*p*.	97.5-*p*.	median	2.5-*p*.	97.5-*p*.	median	2.5-*p*.	97.5-*p*.	median	2.5-*p*.	97.5-*p*.	median	2.5-*p*.	97.5-*p*.	median	2.5-*p*.	97.5-*p*.
CA (µmol/l)	0.117	0.007	0.283	0.085	0.007	0.655	0.034	0.011	0.496	0.040	0.002	0.599	0.055	0.002	0.808	0.048	0.002	1.210
GCA (µmol/l)	0.515	0.093	3.959	0.890	0.094	3.662	0.679	0.068	2.195	0.388	0.031	2.439	0.213	0.028	1.248	0.384	0.086	2.357
TCA (µmol/l)	0.287	0.046	1.469	0.193	0.016	2.253	0.161	0.014	0.798	0.055	0.000	0.532	0.031	0.000	0.355	0.056	0.007	0.200
CDCA (µmol/l)	0.159	0.007	0.533	0.274	0.001	1.214	0.139	0.000	1.507	0.128	0.000	1.277	0.219	0.000	1.810	0.036	0.000	0.729
GCDCA (µmol/l)	1.487	0.312	5.179	3.144	0.115	10.736	2.070	0.238	6.207	1.400	0.160	5.007	1.040	0.113	4.075	1.173	0.259	3.592
TCDCA (µmol/l)	1.310	0.078	3.084	0.717	0.026	3.856	0.491	0.041	1.255	0.176	0.018	1.180	0.131	0.011	0.990	0.122	0.024	0.481
DCA (µmol/l)	0.000	0.000	0.002	0.000	0.000	0.865	0.035	0.000	0.483	0.032	0.000	0.725	0.109	0.000	1.100	0.330	0.008	1.155
GDCA (µmol/l)	0.000	0.000	0.471	0.000	0.000	0.842	0.065	0.000	1.232	0.027	0.000	1.142	0.088	0.000	1.072	0.229	0.004	1.272
TDCA (µmol/l)	0.000	0.000	0.005	0.000	0.000	0.188	0.011	0.000	0.315	0.005	0.000	0.227	0.009	0.000	0.215	0.031	0.000	0.255
LCA (µmol/l)	0.000	0.000	0.001	0.000	0.000	0.028	0.003	0.000	0.063	0.000	0.000	0.053	0.001	0.000	0.045	0.000	0.000	0.019
GLCA (µmol/l)	0.000	0.000	0.008	0.000	0.000	0.045	0.005	0.000	0.071	0.001	0.000	0.066	0.003	0.000	0.079	0.002	0.000	0.056
TLCA (µmol/l)	0.000	0.000	0.002	0.000	0.000	0.007	0.002	0.000	0.014	0.000	0.000	0.020	0.001	0.000	0.015	0.002	0.000	0.008
UDCA (µmol/l)	0.010	0.000	0.157	0.031	0.001	0.694	0.033	0.000	0.317	0.018	0.000	0.373	0.057	0.000	0.618	0.043	0.000	0.678
GUDCA (µmol/l)	0.003	0.000	0.170	0.183	0.007	1.225	0.165	0.000	1.272	0.102	0.002	0.907	0.118	0.000	0.945	0.092	0.010	1.532
TUDCA (µmol/l)	0.003	0.000	0.052	0.017	0.001	0.164	0.016	0.000	0.120	0.006	0.000	0.079	0.007	0.000	0.107	0.007	0.000	0.159
total BA (µmol/l)	5.185	1.824	10.675	6.713	0.549	18.610	4.834	0.548	11.016	3.104	0.447	10.619	2.869	0.494	8.064	2.977	0.939	8.473
total primary (µmol/l)	5.163	1.786	10.447	5.419	0.443	17.736	4.441	0.463	9.798	2.516	0.385	8.759	2.035	0.277	6.842	2.310	0.656	5.770
total secondary (µmol/l)	0.002	0.000	0.473	0.006	0.000	1.628	0.130	0.001	1.862	0.093	0.000	1.976	0.268	0.000	2.157	0.387	0.008	2.297
total tertiary (µmol/l)	0.041	0.001	0.273	0.222	0.021	1.845	0.224	0.009	1.678	0.149	0.006	1.272	0.204	0.000	1.477	0.149	0.028	2.003
total conjugated (µmol/l)	4.643	1.257	10.296	5.617	0.413	17.974	4.291	0.514	10.603	2.514	0.356	9.525	1.990	0.268	7.053	2.275	0.596	6.918
total unconjugated (µmol/l)	0.320	0.039	0.702	0.464	0.039	2.155	0.305	0.019	2.427	0.289	0.027	2.236	0.569	0.028	3.117	0.170	0.027	1.820
total glycine-conjugated (µmol/l)	2.326	0.539	6.875	4.589	0.295	14.364	3.188	0.370	9.166	2.158	0.301	8.358	1.716	0.222	5.977	1.994	0.471	6.496
total taurine-conjugated (µmol/l)	2.042	0.144	3.899	0.981	0.053	5.711	0.658	0.072	1.958	0.288	0.033	1.717	0.214	0.020	1.452	0.229	0.036	0.935
proportion primary (%)	98.96	89.94	99.95	94.05	61.08	99.56	88.76	67.70	99.34	86.24	49.61	99.61	76.00	33.63	99.60	73.66	37.99	98.60
proportion secondary (%)	0.04	0.00	7.27	0.12	0.00	24.42	6.07	0.02	23.72	4.35	0.00	45.21	11.29	0.00	57.11	14.16	0.22	45.13
proportion tertiary (%)	0.89	0.04	2.90	5.42	0.30	25.94	5.21	0.60	18.32	5.47	0.25	25.57	7.73	0.03	34.88	7.18	0.74	33.94
primary to secondary ratio	2759.51	61.99	63162.14	805.41	3.43	68064.96	14.44	3.02	9357.10	19.61	1.14	35755.57	7.05	0.63	17856.04	5.64	0.89	453.11
proportion conjugated (%)	93.00	68.11	99.17	91.56	51.11	98.92	94.01	61.99	98.36	87.83	44.19	99.01	76.73	26.02	97.69	93.23	47.83	99.16
proportion unconjugated (%)	7.00	0.83	31.89	8.44	1.08	48.89	5.99	1.64	38.01	12.17	0.99	55.81	23.27	2.10	74.86	6.77	0.84	52.17
proportion glycine-conjugated (%)	59.38	26.73	84.46	67.70	39.93	90.31	72.45	52.80	85.15	72.83	36.06	92.55	65.64	22.06	89.23	79.13	40.33	95.53
proportion taurine-conjugated (%)	34.25	6.82	67.35	22.84	2.25	42.11	16.27	5.02	28.67	11.24	1.74	31.87	8.03	1.12	31.68	7.90	1.43	22.28
glycine to taurine ratio	1.61	0.46	15.06	2.99	1.19	28.43	4.76	2.29	14.49	6.14	1.88	31.41	7.89	1.76	49.01	11.40	2.21	57.51

*CA* cholic acid, *CDCA* chenodeoxycholic acid, *DCA* deoxycholic acid, *LCA* lithocholic acid, *UDCA* ursodeoxycholic acid, *G* Glycine-, *T* Taurine-

Median total BA were higher in young children compared to adults with a peak at 6–23 months (6.713 µmol/l in 6–23-month-olds vs. 2.869 µmol/l in 12–19-year-olds). Furthermore, the median proportion of primary BA decreased by age from 99.0% in infants to 73.7% in adults, while secondary and tertiary BA increased from 0.04% to 14.2% and 0.9% to 7.2%. To reduce the influence of tertiary BA, as mainly based on ursodeoxycholic acid therapy, on BA interpretation, a further ratio of primary to secondary BA was calculated, showing a decrease from 2759.5 in the youngest to 5.6 in the oldest age group. The BA conjugates showed a decrease of taurine-conjugated BA and increase of glycine-conjugated BA by age leading to an increase in the glycine to taurine ratio from 1.6 in 0–5-months-olds to 11.4 in adults (Table 2).

RefineR could only be applied in the 12–19-year-old age group due to the limited sample size, and we therefore only used this age group to validate the established guidance values. 14 of the 15 bile acids’ guidance values were within the 95% confidence interval of the refineR-generated upper reference limits established for the 12–19-year-old age group. Therefore, we conclude, that for classification of pathological results our guidance values can be used.

### Bile acids in gastrointestinal, hepatic and biliary disorders

Total BA and the proportions of primary, secondary and tertiary BA are ranked in Fig. 1c, revealing highest abundance in acute liver failure, liver cirrhosis, PFIC and Alagille syndrome. Next, dedicated profiles were analyzed in the following paragraphs.

### Bile acid profiles in gastrointestinal disorders

**Fig. 2:**
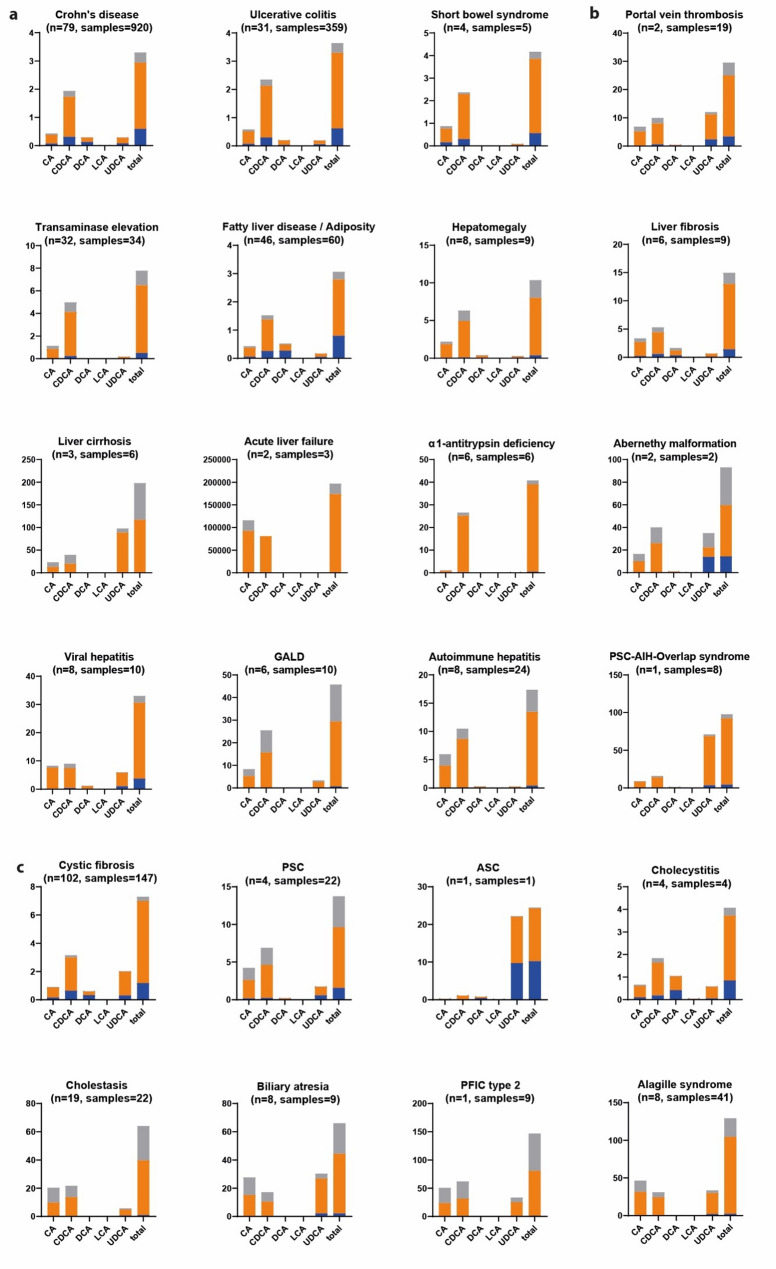
Bile acid profiles of gastrointestinal, hepatic and biliary diseases. CA = cholic acid, CDCA = chenodeoxycholic acid, DCA = deoxycholic acid, LCA = lithocholic acid, UDCA = ursodeoxycholic acid, G = Glycine-, T = Taurine, n = number of patients, samples = number of samples. Displayed are different diseases with their respective median bile acid profile assigned to. **a** gastrointestinal, (**b**) hepatic and (**c**) biliary diseases. orange = glycine-conjugated, grey = taurine-conjugated, blue = unconjugated BAs. Created with Graphpad Prism and Adobe Illustrator software

**Fig. 3:**
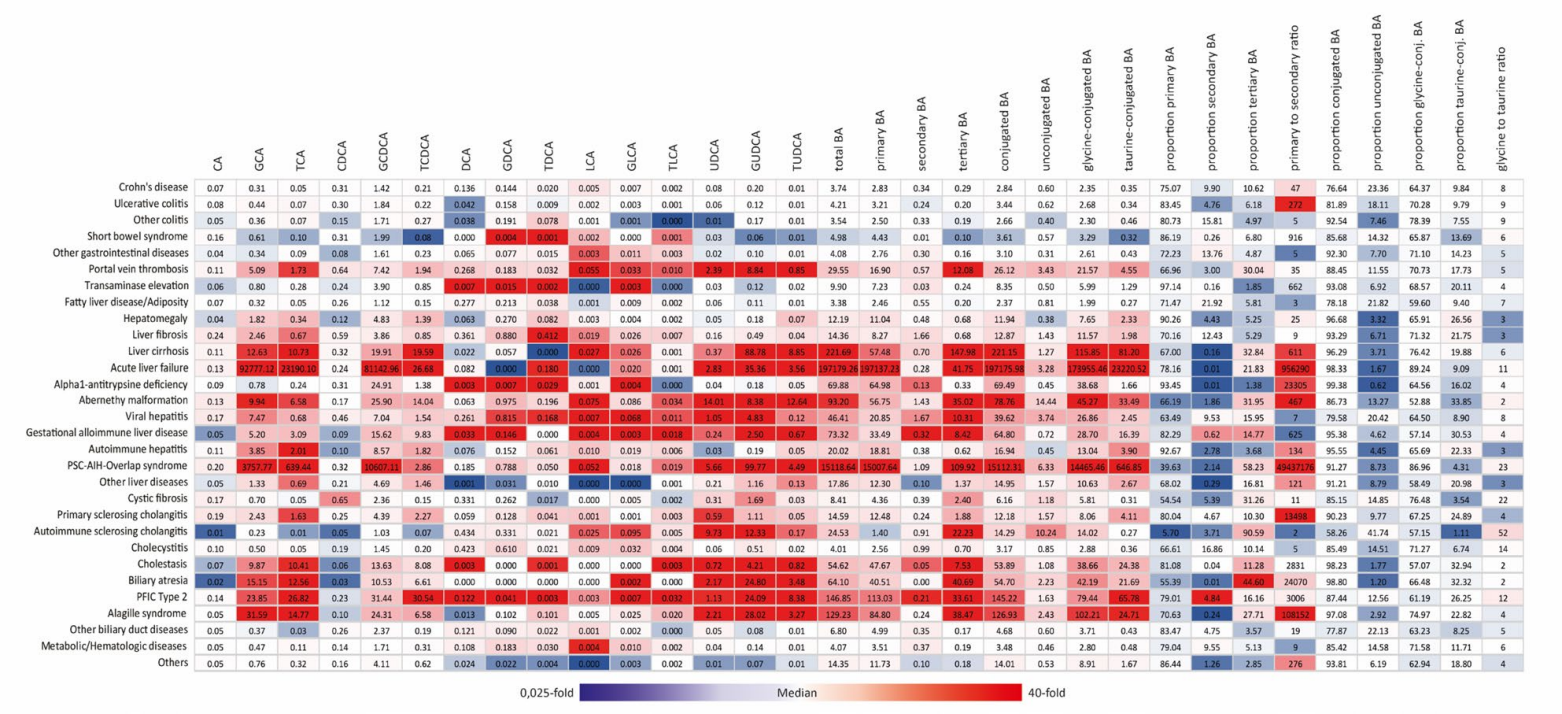
Heat map of altered bile acids in gastrointestinal, hepatic and biliary diseases. CA = cholic acid, CDCA = chenodeoxycholic acid, DCA = deoxycholic acid, LCA = lithocholic acid, UDCA = ursodeoxycholic acid, G = Glycine-, T = Taurine-, displayed in µmol/ml, proportions are displayed as %, ratios are displayed as numbers. The shown values are the median of the respective disease and BA. The colour coding is based on the relative increase or decrease compared to the median value of the corresponding age group. Red = increased compared to the median of the corresponding age group, blue = decreased compared to the median of the corresponding age group. Created with Adobe Illustrator software

920 samples from 79 patients with *Crohn’s disease* (median age = 15 years) were included. Although the median BA profile remained below the established cut-off values, median values of primary, secondary and tertiary BA were slightly increased (e.g., LCA being 5-fold higher compared to the median). 251 (27.7%) of CD patients’ samples showed at least one altered BA or ratio that exceeded the cut-off value (Figs. 2a and 3).

Similarly, samples (*n* = 359) from patients with *ulcerative colitis* showed a slight increase in the median levels of primary, secondary, and tertiary BA. However, the median value of DCA was decreased by 60% when compared to the median of the reference group. Notably, 111 (30.9%) of these samples exhibited pathological BA profiles, which means that they surpassed the cut-off values of at least one bile acid.

We further divided IBD patients into two groups based on disease activity: an active disease state (*n* = 555 samples) and remission (*n* = 355 samples), using calprotectin levels as indicator (cut off = 150 µg/g). The total BA levels did not differ significantly between these groups. However, we observed a statistically significant 25%-increase in primary BA in patients with active IBD (*p* = 0.0041). Additionally, the median values of secondary BA were markedly lower (0.40 vs. 0.09µmol/l, *p* < 0.0001), levels of glycine-conjugated BA higher (1.82 vs. 2.16 µmol/l, *p* = 0.0036) and proportion of unconjugated BA lower (24.0% vs. 18.3%, *p* < 0.0001) in patients with active disease.

Next, we compared the BA profiles of IBD patients with and without PSC. Among the IBD-PSC patients analyzed (*n* = 3 patients, 21 samples), all exhibited elevated levels of taurocholic acid (TCA), with two also showing increased total primary BA. The degree of TCA elevation was highly heterogeneous, ranging from 22% to 1553% above the established cut-off value, but markedly higher than the values of patients with IBD only. Additionally, secondary BA of the two patients with increased primary BA, were reduced by 10- and 100-fold compared to the median.

### Bile acid profiles in hepatic diseases

The disease-specific bile acid profiles of the included hepatic diseases are displayed in Figs. 2b and 3. Patients with *fatty liver disease* (*n* = 46, median age = 13.6 years) generally displayed normal bile acid levels. In contrast, those with *liver fibrosis* (*n* = 6 patients with 9 samples, median age = 14.7 years) showed moderately elevated levels of total primary BA (median = 8.27 µmol/l, 4/6 above the cut-off) and secondary BA (median = 1.66 µmol/l, 2/6 above the cut-off). Additionally, taurine conjugates showed a nine-fold increase, while total BA were only increased by five times compared to the median. This resulted in low glycine to taurine ratios. In patients with *cirrhosis* (*n* = 3 patients with 6 samples, median age = 6.3 years), primary BA were markedly elevated (median = 57.48 µmol/l, 3/3 above the cut-off), while secondary BA remained within normal ranges.

While these diseases primarily reflect chronic liver damage, we also analyzed the BA profiles of patients with acute liver damage. In *acute liver failure* (*n* = 2 patients with 3 samples) markedly elevated levels of primary (conjugated) BA were found, whereas secondary BA and unconjugated primary BA remained nearly normal. Similarly, in patients with *viral hepatitis* (*n* = 8 patients, with 10 samples), primary bile acid levels exceeded the established cut-off value, although less than observed in acute liver failure (median = 21 µmol/l vs. 197137 µmol/l). Patients with *autoimmune hepatitis* (8 patients, 24 samples) exhibited heterogeneous BA profiles, with some patients showing markedly elevated levels of conjugated primary BA (5/8).

To further investigate the relationship between liver dysfunction and alterations in BA profiles, we analyzed serum levels of liver enzyme GOT (AST) in *n* = 490 patients, using average values when multiple samples per patient were available. Correlation analyses with individual bile acids revealed that unconjugated primary BA showed no significant associations, whereas conjugated primary BA exhibited strong positive correlations with GOT levels (*r* = 0.42 to 0.59, *p* < 0.0001 for all). Notably, taurine-conjugated primary BA correlated more strongly than glycine-conjugated counterparts. Correspondingly, the total concentration of taurine-conjugates demonstrated a stronger correlation with GOT (*r* = 0.55, *p* < 0.0001) compared to that of glycine-conjugates (*r* = 0.38, *p* < 0.0001). Additionally, the relative proportion of glycine-conjugates was negatively correlated with GOT levels (*r*=–0.41, 0.0001).

In the metabolic disorder *alpha1-antitrypsin deficiency* (*n* = 6, median age = 11 months), patients exhibited elevated levels of total primary (conjugated) BA, while unconjugated and secondary BA remained within normal ranges.

All infants with *Gestational alloimmune liver disease* (*n* = 6, median age = 3 months) showed elevated median levels of primary and conjugated BA. Additionally, TLCA was increased in five out of six patients.

Compared to patients with *autoimmune hepatitis* without PSC (*n* = 8, median age = 12 years), a patient with *Overlap syndrome* showed a higher proportion of glycine-conjugated BA. In autoimmune hepatitis patients, the primary conjugated BA were more likely to be elevated.

### Bile acid profiles in bile pathologies

The disease-specific BA profiles of the included biliary diseases are displayed in Figs. 2c and 3 and summarized briefly in the following.

In 102 patients with *Cystic fibrosis* (median age = 20 years), glycine-conjugated and tertiary BA were elevated most frequently (median of glycine-conjugates = 5.81 µmol/l, 45/102 above the cut-off).

Other cholestatic diseases such as *primary sclerosing cholangitis* (*n* = 4, median age = 11 years) presented themselves heterogenous. The proportion of taurine-conjugates was increased compared to the median in three out of four patients. But, in one patient with *autoimmune sclerosing cholangitis* (age = 13 years) a decreased proportion of taurine-conjugated BA and an elevated glycine to taurine ratio was found as well as the highest proportion of tertiary BA (90.6%).

In neonates and infants with *cholestasis* (*n* = 19, median age = 0.5 months) and *biliary atresia* (*n* = 8, median age = 1 month) the same pattern in elevated BA was found with elevated primary (conjugated) and tertiary BA. Furthermore, eight out of 19 cholestasis patients showed an increased proportion of taurine-conjugates. In five out of nine samples of biliary atresia, it showed an elevation of conjugated BA, especially taurine-conjugates with a decrease in the glycine to taurine ratio. The differences in BA profiles in biliary atresia before and after intervention are shown in Supplementary Fig. 2. After intervention (*n* = 1), the glycine to taurine ratio and unconjugated BA increased.

Patients with *Alagille syndrome* (*n* = 8, median age = 3 years) showed an increase of conjugated primary BA with a 7489-fold increase in the ratio of primary to secondary BA and both glycine- and taurine-conjugates being significantly elevated (32- and 38-fold). They had the second highest conjugation ratio compared to all diagnoses. Tertiary BA were above the cut-off in seven out of eight patients.

To evaluate the link between gamma-GT levels and bile acids, two groups were compared (elevated gamma-GT levels > 20 U/L, *n* = 653 vs. normal gamma-GT levels, *n* = 1255). While secondary BA did not differ significantly between the two groups, the total primary BA concentration was nearly twice as high in patients with elevated gamma-GT levels (4.5 µmol/l vs. 2.4 µmol/l, *p* < 0.0001).

### Bile acid profiles by age group

Next, disease groups with *n* ≥ 10 cases were divided in two age groups, below and over one year. In all analyzed groups (Crohn’s disease, cystic fibrosis, transaminase elevation and cholestasis) an increased glycine- to taurine- conjugation ratio was observed in over one year olds. In Crohn’s disease, cystic fibrosis and transaminase elevation a further increase of unconjugated BA could be shown. (Supplementary Fig. 2).

## Discussion

In this study, a large dataset of routine BA measurements from children with various gastrointestinal, hepatic and biliary diseases was analyzed with the potential to serve as a framework with immediate benefits for clinical practice and future studies in the field.

First, we provide BA guidance values in pediatrics through prior filtering. Due to the small sample size in some age groups advanced data-driven methods for reference interval establishments like refineR [18] cannot be applied. However, we exemplarily validated the guidance values for our largest age group (12–19 years) using the refineR algorithm, confirming the guidance values in 14 out of 15 cases. Therefore, we assume that our guidance values for the other age groups might be used for interpretation and to put the results of our diseases groups in context. We provide the 2.5th and 97.5th percentile for all BA components and ratios to aid clinicians classify their measurements as current reference intervals are based on small cohorts and not available for several diseases [10, 16].

We found standard values for total BA to be higher in younger compared to older children. Furthermore, a rise of secondary and glycine-conjugated BA was observed. These findings are in line with previous observations [16].

While patients with IBD appeared to have similar BA profiles compared to healthy controls, we demonstrated that bile acid profiles in patients with active disease exhibit significant differences. Notable variations were observed, particularly in primary BA, conjugation status, and secondary BA, which align with findings from a previous study [10]. However, the total bile acid count remained unaffected, underscoring the advantage of assessing bile acid profiles rather than relying solely on total bile acid levels. The mechanism underlying these changes remains uncertain but alterations in the microbiome may trigger the inflammatory process, reflected by changes in secondary BA [19, 20]. This could potentially enable the detection of microbiome changes that precede inflammation and elevated calprotectin levels. Furthermore, detection and monitoring of primary sclerosing cholangitis in IBD patients might be enabled by detection of increased taurine-conjugated forms in particular, which was also observed by Mousa et al. in 2021 [21]. When comparing IBD-PSC patients to IBD-only, an increase in primary BA and a decrease in secondary BA were observed in the presence of PSC comorbidity, resulting in a corresponding rise in the primary-to-secondary BA ratio. In contrast, in the 2024 study by Leibovitzh et al., both primary and secondary BA showed an increase, however, the primary-to-secondary BA ratio was in line with our study [22]. Next, longitudinal studies in IBD patients are necessary to further elucidate the underlying mechanisms and clinical utility of these findings.

In chronic liver diseases, changes in the BA profile can be identified in relation to the stage of the disease [23]. However, it is not only the total BA level that is relevant since for instance primary and secondary BA are differently affected by the disease progression and localization but also the glycine-to-taurine ratio shows alterations [17, 24, 25]. BA could therefore provide a less invasive approach for tracking and predicting the progression of chronic liver disease and even for assignment of origin [1, 26]. In liver diseases, it is observed that BA and transaminases are not always elevated to the same extent. This discrepancy is likely due to the fact that both parameters are influenced by a range of complex factors and that BA play a greater role in hepatic metabolism, inflammation and signalling [1].

When examining biliary diseases, the increase in primary conjugated and tertiary BA is particularly striking. This can be explained by the cholestasis and the intake of ursodeoxycholic acid, but the extend of altered BA in these patients might be used as a surrogate biomarker in these diseases [12, 27]. Especially in children with Alagille and PFIC with new therapies influencing the bile metabolism, monitoring of BA is of special interest [13–15, 27, 28].

Furthermore, we have identified a method to distinguish the BA profiles of patients undergoing ursodeoxycholic acid therapy, achieving excellent AUC with a cut-off of 2.322 µmol/l. This simple differentiation criterion could be utilized for monitoring patient compliance and enhancing therapy planning. It is important to note that the intake of UDCA also affects the overall BA profile, influencing factors such as the conjugation status [21], and increases the proportion of LCA [29].

Our study is limited by the retrospective design. However, in view of the otherwise very time-consuming and difficult feasibility of such a prospective study in children, this routine data is an excellent opportunity for an initial framework. Future prospective studies should be conducted. Although some disease groups are very small, the entire dataset is large considering a pediatric cohort. However, the data does not include the measurement of newer, microbially modified BA (e.g. iso-/allo-species) which could play a central role in various diseases such as cystic fibrosis, IBD and cholestatic liver disease [30, 31]. 

It would be great to encourage clinicians to test (newer) BA profiles more frequently and collect and merge data on rare diseases to accelerate routine implementation in clinics. In addition, information on the fasting status of the patients was not collected and treatments and extent of each disease were not included.

Summarizing, this work provides a comprehensive framework for BA profiles in pediatric patients with gastrointestinal, hepatic and biliary diseases. This could aid both, clinicians and researchers in the field. The clinical impact of BA as biomarkers should be further investigated.

## Supplementary Information

Supplementary Material 1.

## Data Availability

Anonymized data will be available from the corresponding author on reasonable request.

## Abbreviations

BA: Bile acid
CA: Cholic acid
CDCA: Chenodeoxycholic acid
DCA: Deoxycholic acid
LCA: Lithocholic acid
UDCA: Ursodeoxycholic acid
G: Glycine-
T: Taurine-
AUC: Area under the curve
CD: Crohn’s disease
IBD: Inflammatory bowel disease
PSC: Primary sclerosing cholangitis
