# Supplementary material for: Serum bile acid profiles in pediatric gastrointestinal, hepatic and biliary diseases
Source: Mol Cell Pediatr. 2025 Nov 28;12:23. doi: 10.1186/s40348-025-00211-2 (PMC12660533; doi:10.1186/s40348-025-00211-2)
Supplement: Supplementary file 1 — Supplementary Material 1. [file 40348_2025_211_MOESM1_ESM.docx]

**Supplementary Information to:**

**Serum bile acid profiles in pediatric gastrointestinal, hepatic and biliary diseases**

Katja Linz^1*^, Felix Wachter^1*^, MD, Merle Claßen^1^, MD, PhD, Jakob Zierk^1^, MD, Theresa Voggenreiter^1^, Alexander Schnell^1^, MD, Henriette Grieshaber Bouyer Mandelbaum^1^, MD, Joachim Woelfle^1^, MD, Ferdinand Knieling^1^, MD, PhD, André Hoerning^1^, MD, Manfred Rauh^1^, PhD, Adrian P. Regensburger^1#^, MD, PhD

^1^Department of Pediatrics and Adolescent Medicine, Friedrich-Alexander-University (FAU) Erlangen-Nürnberg, Erlangen, Germany

* Authors contributed equally, # Corresponding author

Adrian P. Regensburger

Department of Pediatrics and Adolescent Medicine

Friedrich-Alexander-University (FAU) Erlangen-Nürnberg

Loschgestr. 15

91054 Erlangen, Germany

ki-forschung@uk-erlangen.de

+49 9131 85 33118

**TABLE OF CONTENTS**

[Supplementary Table 1 – Gastrointestinal, hepatic and biliary diseases 3](#_Toc211940041)

[Supplementary Figure 1 – Biliary atresia before and after intervention 4](#_Toc211940042)

[Supplementary Figure 2 – Age groups </>1 year 5](#_Toc211940043)

## Supplementary Table 1 – Gastrointestinal, hepatic and biliary diseases

| Group | Diagnosis | Samples | Patients | Median age | Cut-off group |
| --- | --- | --- | --- | --- | --- |
| Hepatic | Abernethy malformation | 2 | 2 | 5 years | 2-5 years |
| Hepatic | Acute liver failure | 3 | 2 | 8 years | 6-11 years |
| Biliary | Alagille syndrome | 41 | 8 | 3 years | 2-5 years |
| Hepatic | Alpha1-antitrypsin deficiency | 6 | 6 | 11 months | 6-23 months |
| Hepatic | Autoimmune hepatitis | 24 | 8 | 12 years | 12-19 years |
| Biliary | Autoimmune sclerosing cholangitis | 1 | 1 | 13 years | 12-19 years |
| Biliary | Biliary atresia | 9 | 8 | 1 month | 0-5 months |
| Biliary | Cholecystitis | 4 | 4 | 16,5 years | 12-19 years |
| Biliary | Cholestasis | 22 | 19 | 0,5 months | 0-5 months |
| Gastrointestinal | Crohn's disease | 920 | 79 | 15 years | 12-19 years |
| Biliary | Cystic Fibrosis | 147 | 102 | 20 years | >19 years |
| Hepatic | Fatty liver disease/Adiposity | 60 | 46 | 14 years | 12-19 years |
| Hepatic | Gestational alloimmune liver disease | 10 | 6 | 3 months | 0-5 months |
| Hepatic | Hepatomegaly | 9 | 8 | 12 years | 12-19 years |
| Hepatic | Liver cirrhosis | 6 | 3 | 6,5 years | 6-11 years |
| Hepatic | Liver fibrosis | 9 | 6 | 14 years | 12-19 years |
| Others | Metabolic/Hematologic diseases | 39 | 23 | 7 years | 6-11 years |
| Biliary | Other biliary duct diseases | 14 | 12 | 9 years | 6-11 years |
| Gastrointestinal | Other colitis | 11 | 8 | 12 years | 12-19 years |
| Gastrointestinal | Other gastrointestinal diseases | 46 | 45 | 6,5 years | 6-11 years |
| Hepatic | Other liver diseases | 20 | 13 | 15,5 years | 12-19 years |
| Others | Others | 37 | 32 | 2 years | 2-5 years |
| Biliary | PFIC Type 2 | 9 | 1 | 8 months | 6-23 months |
| Hepatic | Portal vein thrombosis | 19 | 2 | 8 years | 6-11 years |
| Biliary | Primary sclerosing cholangitis | 22 | 4 | 11 years | 6-11 years |
| Hepatic | PSC-AIH Overlap syndrome | 8 | 1 | 16,5 years | 12-19 years |
| Gastrointestinal | Short bowel syndrome | 5 | 4 | 1 month | 6-23 months |
| Hepatic | Transaminase elevation | 34 | 32 | 1,5 months | 6-23 months |
| Gastrointestinal | Ulcerative colitis | 359 | 31 | 15 years | 12-19 years |
| Hepatic | Viral hepatitis | 10 | 8 | 6,5 years | 6-11 years |

Supplementary Table 1 – Gastrointestinal, hepatic and biliary diseases

Overview of the different diagnosis groups with their sample and patient sizes. The cut-off group is the reference group in which the median age of the affected group is situated.

**Supplementary Figure 1 – Biliary atresia before and after intervention**

**
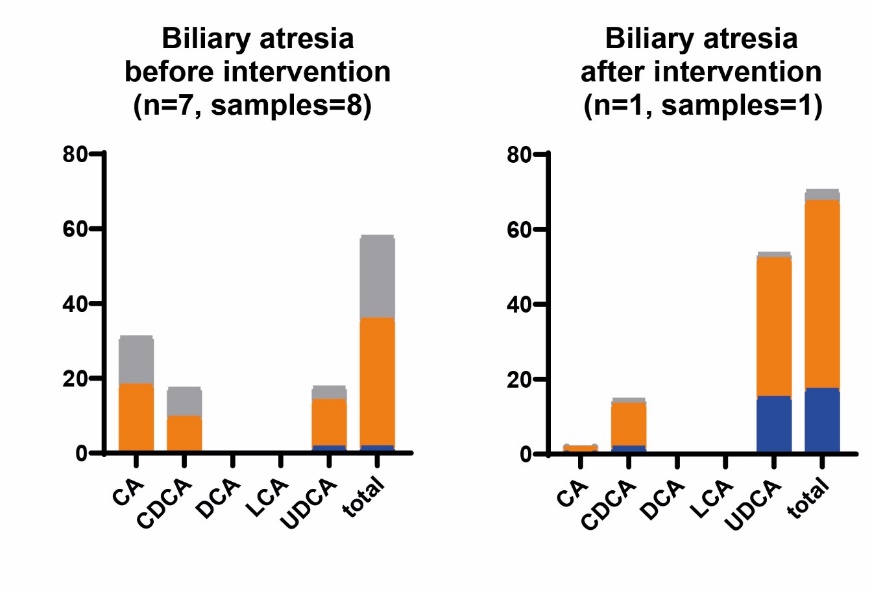
**

**Supplementary Figure 1– Biliary atresia before and after intervention**

CA = cholic acid, CDCA = chenodeoxycholic acid, DCA = deoxycholic acid,

LCA = lithocholic acid, UDCA = ursodeoxycholic acid, G = Glycine-, T = Taurine, n = number of patients, samples = number of samples

Displayed are all biliary atresia patients with their respective median bile acid profile before and after intervention.

orange = glycine-conjugated, grey = taurine-conjugated, blue = unconjugated BAs

Created with Graphpad Prism and Adobe Illustrator software

## Supplementary Figure 2 – Age groups </>1 year


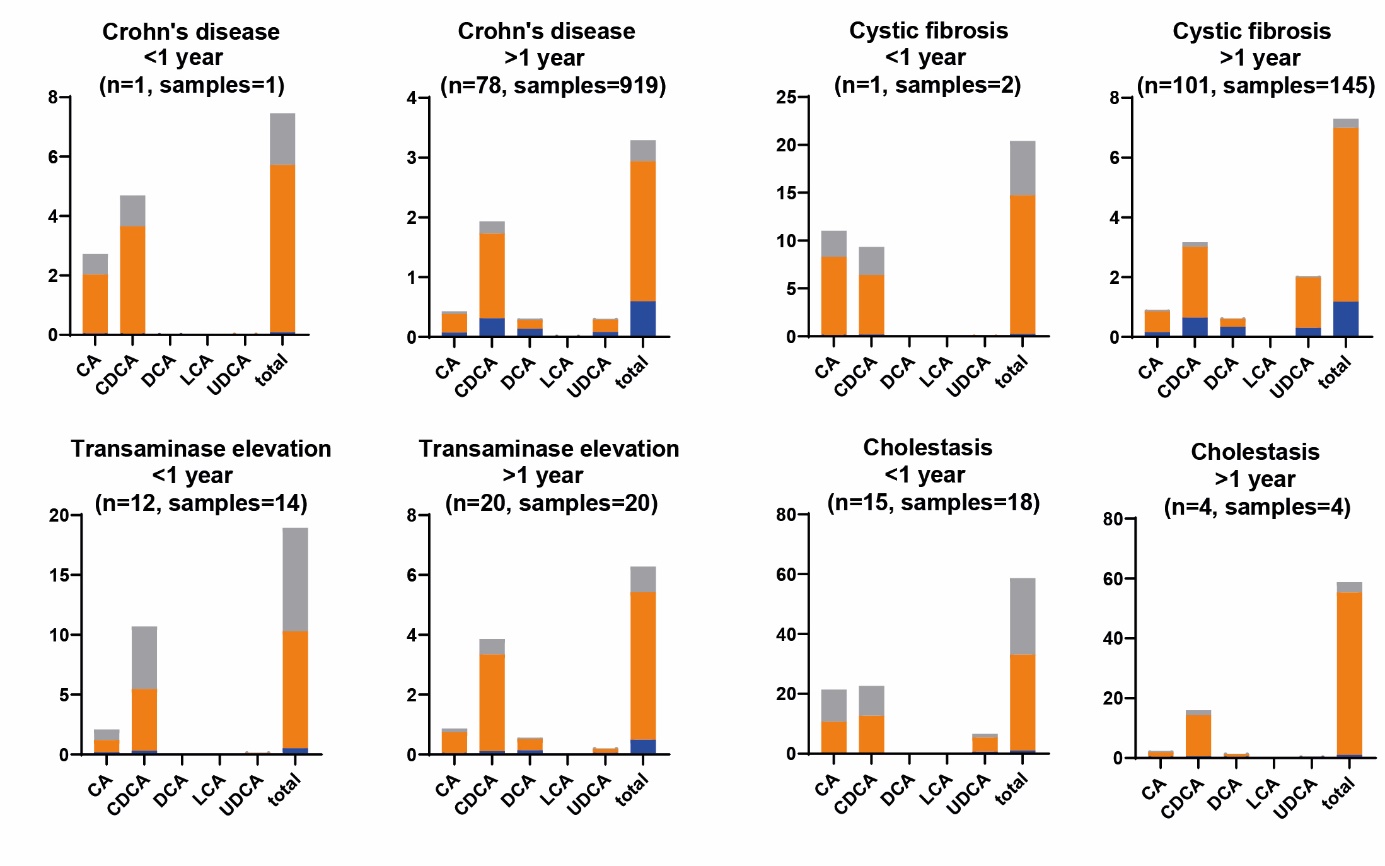


**Supplementary Figure 2 - Age groups </>1 year**

Figure 2: blue = unconjugated, orange = glycine, grey = taurine

CA = cholic acid, CDCA = chenodeoxycholic acid, DCA = deoxycholic acid,

LCA = lithocholic acid, UDCA = ursodeoxycholic acid, G = Glycine-, T = Taurine, n = number of patients, samples = number of samples

Displayed are all diseases with n ≥10, divided by age groups under and over 1 year with their respective median bile acid profile. Ulcerative Colitis and Fatty liver/Adiposity are not shown, because there were no patients under 1 year of age.

orange = glycine-conjugated, grey = taurine-conjugated, blue = unconjugated BAs

Created with Graphpad Prism and Adobe Illustrator software
